# InvL, an Invasin-Like Adhesin, Is a Type II Secretion System Substrate Required for Acinetobacter baumannii Uropathogenesis

**DOI:** 10.1128/mbio.00258-22

**Published:** 2022-05-31

**Authors:** Clay D. Jackson-Litteken, Gisela Di Venanzio, Nguyen-Hung Le, Nichollas E. Scott, Bardya Djahanschiri, Jesus S. Distel, Evan J. Pardue, Ingo Ebersberger, Mario F. Feldman

**Affiliations:** a Department of Molecular Microbiology, Washington University School of Medicine, St. Louis, Missouri, USA; b Department of Microbiology and Immunology, University of Melbourne at the Peter Doherty Institute for Infection and Immunity, Parkville, VIC, Australia; c Applied Bioinformatics Group, Institute of Cell Biology and Neuroscience, Goethe University, Frankfurt am Main, Germany; d Senckenberg Biodiversity and Climate Research Centre (S-BIKF), Frankfurt am Main, Germany; e LOEWE Center for Translational Biodiversity Genomics (TBG), Frankfurt am Main, Germany; University of Pittsburgh

**Keywords:** *Acinetobacter*, adhesin, infection, invasin, pathogenesis, type II secretion system, urinary tract infection, virulence

## Abstract

Acinetobacter baumannii is an opportunistic pathogen of growing concern, as isolates are commonly multidrug resistant. While A. baumannii is most frequently associated with pulmonary infections, a significant proportion of clinical isolates come from urinary sources, highlighting its uropathogenic potential. The type II secretion system (T2SS) of commonly used model Acinetobacter strains is important for virulence in various animal models, but the potential role of the T2SS in urinary tract infection (UTI) remains unknown. Here, we used a catheter-associated UTI (CAUTI) model to demonstrate that a modern urinary isolate, UPAB1, requires the T2SS for full virulence. A proteomic screen to identify putative UPAB1 T2SS effectors revealed an uncharacterized lipoprotein with structural similarity to the intimin-invasin family, which serve as type V secretion system (T5SS) adhesins required for the pathogenesis of several bacteria. This protein, designated InvL, lacked the β-barrel domain associated with T5SSs but was confirmed to require the T2SS for both surface localization and secretion. This makes InvL the first identified T2SS effector belonging to the intimin-invasin family. InvL was confirmed to be an adhesin, as the protein bound to extracellular matrix components and mediated adhesion to urinary tract cell lines *in vitro*. Additionally, the *invL* mutant was attenuated in the CAUTI model, indicating a role in Acinetobacter uropathogenesis. Finally, bioinformatic analyses revealed that InvL is present in nearly all clinical isolates belonging to international clone 2, a lineage of significant clinical importance. In all, we conclude that the T2SS substrate InvL is an adhesin required for A. baumannii uropathogenesis.

## INTRODUCTION

Acinetobacter spp. belonging to the Acinetobacter calcoaceticus–Acinetobacter baumannii (ACB) complex are opportunistic pathogens that cause diverse infections, with A. baumannii representing the most common species associated with human infection (1–3). While A. baumannii infections can be both community and hospital acquired, nosocomial infections in critically ill patients are common, with mortality rates reported as high as 84.3% in individuals requiring mechanical ventilation (4–7). These high mortality rates can be largely attributed to increasing multidrug resistance among A. baumannii isolates (6, 8). In fact, A. baumannii exhibits the highest prevalence of multidrug resistance among Gram-negative pathogens, leading the World Health Organization to classify the bacterium as a highest priority pathogen for research and development of new treatments (8, 9). The respiratory tract is the most common site of infection by A. baumannii, with approximately 40% of the isolates derived from pulmonary infections (2, 3, 10, 11). It should be noted though that 20% of A. baumannii isolates come from urinary tract infections (UTIs), highlighting the bacterium’s uropathogenic capacity (10, 11). However, despite the public health relevance of A. baumannii as a uropathogen, few virulence factors required specifically for UTI have been defined (10).

Bacterial virulence relies on the interaction of bacterially derived proteins with the infected host. Gram-negative bacteria have evolved to encode complex secretion systems to transport proteins across the bacterial envelope (12). The type II secretion system (T2SS) is widely distributed in Gram-negative bacteria and has been implicated in the virulence of several pathogens (13–15). T2SS effectors are translocated from the cytoplasm to the outer membrane or into the extracellular milieu in two steps. First, the effector is transported across the inner membrane into the periplasm via the general secretory (Sec) pathway or the twin arginine translocation (TAT) pathway (16, 17). Second, the effector is extruded through the outer membrane secretin (designated GspD herein) via the assembly of a pseudopilus (18–22). In Acinetobacter, the T2SS is responsible for secretion of several proteins, including the lipases LipA, LipH, and LipAN, the protease CpaA, and the γ-glutamyltransferase GGT (23–28). For A. baumannii strain ATCC 17978, a meningitis isolate from 1951, mutation of the T2SS results in attenuation in bacteremia and pneumonia models of infection (23, 29). Additionally, mutation of the T2SS in Acinetobacter nosocomialis strain M2 and A. baumannii strain AB5075, musculoskeletal isolates from 1996 and 2008, respectively, results in decreased bacterial burden in a pneumonia model of infection (24, 28). Furthermore, the Acinetobacter effectors LipAN of strain ATCC 17978 and CpaA of strain M2 are required for full virulence in a pneumonia model, and GGT of strain AB5075 is required for full virulence in a bacteremia model (23, 27, 28). While the T2SS and associated effectors clearly have roles in pathogenesis in multiple models of infection, the potential function of the T2SS in Acinetobacter uropathogenesis has not been investigated. Additionally, T2SS-dependent secretomes can differ significantly between strains, and these dissimilarities can lead to strain-dependent differences in virulence potential mediated by the T2SS (24, 28, 29). Therefore, evaluation of the T2SS in diverse clinical isolates could reveal previously unrecognized effectors that may serve as novel Acinetobacter virulence factors.

Intimate interaction of bacteria with host tissues at the site of infection is often required for pathogenesis. Indeed, adherence to and/or invasion of host epithelial cells, mediated by bacterial adhesins, is an essential process for the virulence of several pathogens (30). Many adhesins are displayed on the bacterial surface in a type V secretion system (T5SS)-dependent manner (31). These T5SSs, also termed autotransporters (ATs), are proteins consisting of two general components, a β-barrel domain, which is inserted into the outer membrane, and a passenger domain, which uses the β-barrel for transportation to the outer surface of the bacteria (32–34). The intimin-invasin family of ATs specifically are adhesins that consist of an N-terminal β-barrel domain and a C-terminal passenger domain containing multiple immunoglobulin (IG)-like domains often capped by a lectin-like domain (35, 36). Intimin is encoded by many pathogens such as Escherichia coli and functions by binding to a type III secretion system (T3SS) effector, translocated intimin receptor (TIR), which is inserted into the host membrane (37, 38). Invasin (InvA), encoded by Yersinia enterocolitica and Yersinia pseudotuberculosis, functions by binding directly to host β1-integrins, leading to a host cytoskeletal rearrangement and subsequent internalization of the bacteria (39, 40). Importantly, deletion of genes encoding intimin-invasin family proteins results in attenuation in animal models of infection (35, 36). Despite this key role that intimin-invasin family proteins play in the pathogenesis of other bacteria, potential homologs have not been identified in Acinetobacter.

In this work, we aimed to interrogate the function of the T2SS in the recent A. baumannii urinary clinical isolate, UPAB1, using a murine catheter-associated UTI (CAUTI) model and define effectors potentially required for virulence (10). In this pursuit, we identify a novel T2SS effector/intimin-invasin family protein important for Acinetobacter uropathogenesis.

## RESULTS

### The UPAB1 T2SS mutant is attenuated in the CAUTI model.

**FIG 1:**
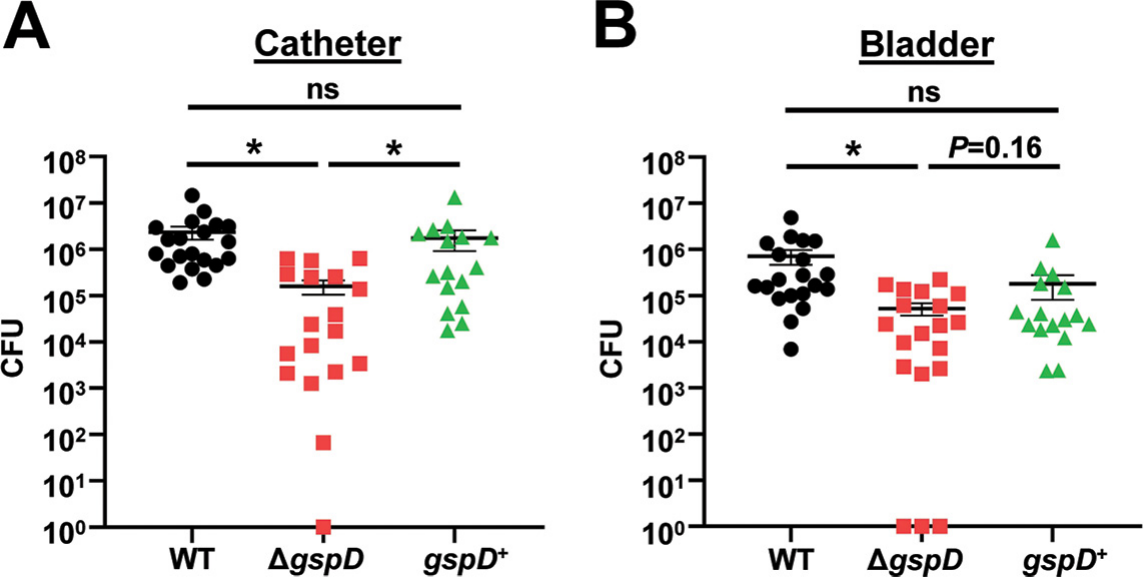
The T2SS is required for full virulence in a murine CAUTI model. Mice were implanted with a catheter followed by transurethral inoculation with UPAB1 WT, Δ*gspD*, or *gspD*^+^ strains. At 24 h postinfection, mice were sacrificed, and bacterial burdens on the catheter (A) and in the bladder (B) were quantified. Shown are results from at least three pooled experiments. Each data point represents an individual mouse, the horizontal line represents the mean, and the standard error of the mean (SEM) is indicated by error bars. *, *P < *0.05; two-tailed Mann-Whitney *U* test; ns, not significant.

To test the role of the T2SS in uropathogenesis, we generated a UPAB1 mutant strain lacking *gspD*, the gene encoding the outer membrane secretin that is essential for T2SS function. This strain was subsequently employed in the murine CAUTI model as previously described (10). Briefly, a small piece of silicon tubing (catheter) was inserted into the urethra, and mice were transurethrally inoculated with wild-type (WT), Δ*gspD*, or complemented (*gspD*^+^) strains. At 24 h postinfection, mice were sacrificed, and bacteria adhered to the catheters (Fig. 1A) and bacterial burdens in the bladders (Fig. 1B) were quantified. The Δ*gspD* mutant exhibited decreased binding to the catheter and colonization of the bladder, with approximately 10-fold fewer CFU recovered relative to WT. The catheter binding phenotype was completely restored by genetic complementation, and the complemented strain exhibited a trend toward higher bladder burden relative to the mutant as the *P* value approached significance. These results indicate that the T2SS plays a role in Acinetobacter uropathogenesis.

### A proteomic analysis identifies a putative T2SS effector with structural similarity to *Yersinia* invasin.

To identify T2SS effectors in UPAB1, we performed a proteomic analysis of the supernatant of WT and Δ*gspD* strains. Proteins with decreased abundance with the mutant strain relative to WT were considered putative T2SS effectors (see Table S1 in the supplemental material). As expected, we identified orthologs of proteins found in previous Acinetobacter T2SS effector screens, including the most differentially identified protein, a 5′-methylthioadenosine (MTA)/*S*-adenosylhomocysteine (SAH) nucleosidase, as well as lipases, a CSLREA domain-containing protein, GGT, and the glycoprotease CpaA (24, 28, 29). However, we additionally identified several proteins not found in previous analyses, several of which were annotated as hypothetical proteins of unknown function.

10.1128/mbio.00258-22.2 TABLE S1 Putative T2SS-dependent secretome of UPAB1. Download Table S1, DOCX file, 0.04 MB. Copyright © 2022 Jackson-Litteken et al. 2022 Jackson-Litteken et al. https://creativecommons.org/licenses/by/4.0/ This content is distributed under the terms of the Creative Commons Attribution 4.0 International license.

**FIG 2:**
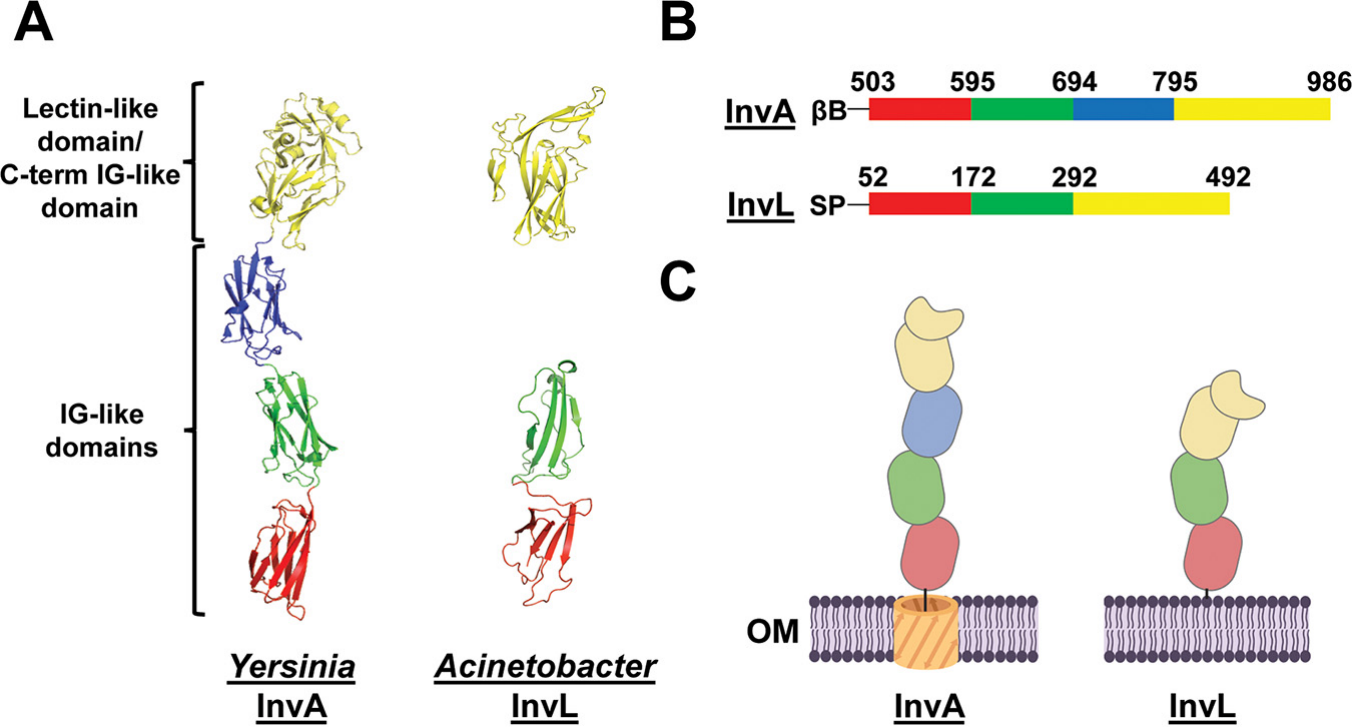
InvL has predicted structural similarity to InvA of *Yersinia*. (A) The crystal structure of the passenger domain of *Yersinia* InvA (PDB entry 1CWV) is shown on the left, and the AlphaFold2 predicted structure of the analogous region of InvL is pictured on the right. (B) Amino acids corresponding to subdomains of the passenger domain of InvA and the predicted homologous region of InvL. (C) Graphic depiction of the structure of InvA and the predicted structure of InvL; created with BioRender.com. Red, green, and blue colors denote individual IG-like domains, and yellow denotes the C-terminal IG-like domain in intimate contact with the lectin-like domain. βB, β-barrel domain; SP, signal peptide; OM, outer membrane.

The most differentially abundant protein of unknown function in the supernatant of WT and Δ*gspD* strains was D1G37_RS04395. D1G37_RS04395 is a 492-amino-acid, 51.92-kDa protein predicted by SignalP 5.0 to contain an N-terminal lipoprotein secretion signal (41). Interestingly, a fold recognition analysis of D1G37_RS04395 with Phyre^2^ revealed the invasin InvA of Y. pseudotuberculosis as the best match (99.7% confidence; 72.0% coverage) (Fig. S1) (42, 43). Submission of D1G37_RS04395 to I-TASSER similarly predicted structural similarity to InvA (2.03 normalized Z score; 77.0% coverage) (44–46). Alphafold2 was then used to generate a model of D1G37_RS04395 to compare to the known structure of InvA (Fig. 2A) (43, 47). D1G37_RS04395 is predicted to have three IG-like domains and is capped with a C-terminal lectin-like domain that is intimately associated with the most C-terminal IG-like domain. This is comparable to the passenger domain of InvA, with the exception that InvA contains an additional IG-like domain (43). A key difference between D1G37_RS04395 and InvA, however, is that InvA is an autotransporter (T5SS) attached to the membrane by an N-terminal β-barrel domain, whereas D1G37_RS04395 is a putative T2SS effector predicted to be attached to the membrane as a lipoprotein (Fig. 2B and C) (35, 36). Of note, we performed alignments of the C-terminal 440 amino acids of InvL and 483 amino acids of InvA using LALIGN software, as these were the regions of predicted homology from our structural analysis. This revealed an overlap of 285 amino acids with 22.5% identity and 51.2% similarity. It should be noted though that the IG-like and lectin domains of the intimin-invasin family fold similarly but have little sequence identity (36, 48). Therefore, extensive similarity at the amino acid sequence level between the proteins was not expected. Overall, given specifically the predicted structural similarity of D1G37_RS04395 to the passenger domain of InvA, we will refer to this protein herein as InvL (invasin-like protein).

10.1128/mbio.00258-22.5 FIG S1 Phyre^2^ predicts that D1G37_RS04395 has structural similarity to *Yersinia* InvA. Results from the Phyre^2^ analysis of the amino acid sequence of full-length D1G37_RS04395 are shown. Download FIG S1, TIF file, 1.1 MB. Copyright © 2022 Jackson-Litteken et al. 2022 Jackson-Litteken et al. https://creativecommons.org/licenses/by/4.0/ This content is distributed under the terms of the Creative Commons Attribution 4.0 International license.

### The T2SS is required for InvL secretion and surface exposure.

**FIG 3:**
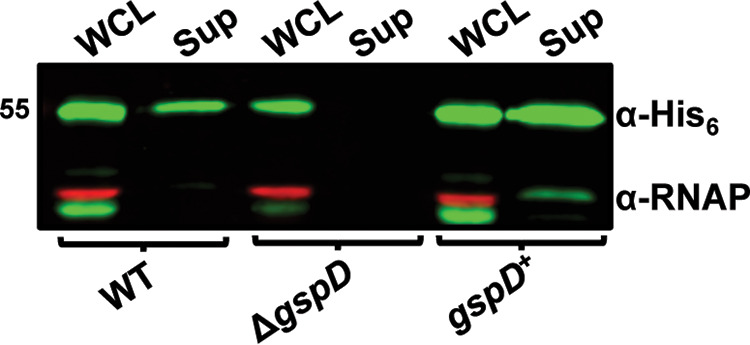
The T2SS is required for InvL secretion. Whole-cell lysate (WCL) and supernatant (Sup) fractions from WT, Δ*gspD*, and *gspD*^+^ UPAB1 cultures harboring pBAV-Apr::*invL*-*his_6_* were probed for InvL (α-His_6_) and RNAP by immunoblotting. Numbers to the left indicate molecular weight in kDa. At least two biological replicates were performed, yielding similar results, and a representative blot from one replicate is shown.

**FIG 4:**
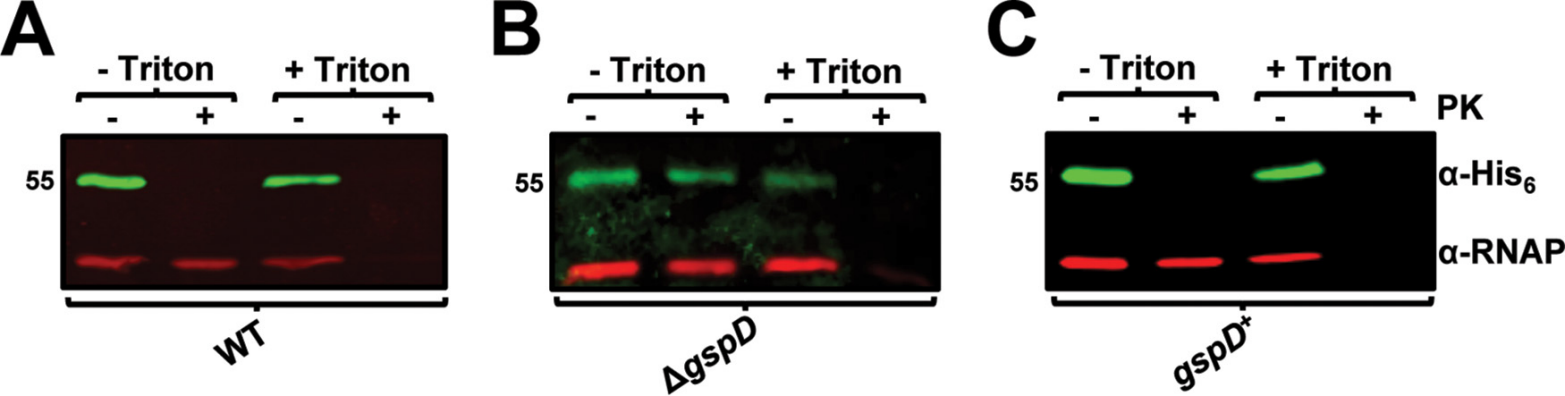
The T2SS is required for InvL surface localization. WT (A), Δ*gspD* (B), and *gspD*^+^ (C) UPAB1 cells harboring pBAV-Apr::*invL*-*his_6_* were treated with proteinase K (PK; +) or left untreated (−) in the presence or absence of Triton X-100. Cells were then probed for InvL (α-His_6_) and RNAP by immunoblotting. Numbers to the left indicate molecular weight in kDa. At least two biological replicates were performed, yielding similar results, and a representative blot from one replicate is shown.

To confirm that secretion of InvL is dependent on a functional T2SS, WT, Δ*gspD*, and *gspD*^+^ strains were transformed with a plasmid expressing *invL* with a His_6_ tag (pBAV-Apr::*invL*-*his_6_*). Whole-cell lysate and supernatant fractions from the bacteria were then assessed by immunoblotting for the presence of InvL-His_6_ (Fig. 3). InvL was found in the supernatant of WT and *gspD*^+^ strains, whereas the Δ*gspD* mutant failed to secrete the protein. This confirmed that InvL is a T2SS substrate. Immunoblot analyses demonstrated that, in addition to being released into the supernatant, InvL was cell associated (Fig. 3). As intimin-invasin family proteins function as surface-localized adhesins, we hypothesized that cell-associated InvL is surface localized. To test this, proteinase K susceptibility assays wherein surface-exposed proteins are degraded and intracellular proteins are left intact were performed with bacteria expressing *invL*-*his_6_*. In WT UPAB1, InvL-His_6_ was completely degraded with proteinase K treatment, whereas the intracellular control, RNA polymerase (RNAP), was not (Fig. 4A). On the contrary, treatment with Triton X-100 and proteinase K resulted in degradation of both InvL-His_6_ and RNAP. These results indicate that InvL is surface localized, consistent with its putative role as an adhesin. Interestingly, proteinase K failed to degrade InvL-His_6_ expressed in the Δ*gspD* mutant, indicating the protein did not reach the surface of the bacteria (Fig. 4B). Alternatively, InvL-His_6_ was readily degraded by proteinase K treatment of *gspD*^+^ (Fig. 4C), demonstrating that the T2SS is required for InvL surface localization.

As InvL is predicted to be a lipoprotein, its identification in the supernatant is somewhat surprising. To further examine the form in which InvL is secreted, insoluble and soluble fractions from the supernatant of WT UPAB1 expressing *invL*-*his_6_* were separated by ultracentrifugation. Immunoblot analysis of these fractions revealed that InvL was primarily found in the insoluble fraction (Fig. S2). This localization could be due to the presence of InvL in either lysis by-products or possibly outer membrane vesicles (OMVs). The physiological relevance of OMVs in Acinetobacter pathobiology remains to be investigated and will be the focus of subsequent work.

10.1128/mbio.00258-22.6 FIG S2 InvL localizes primarily to the insoluble fraction of bacterial supernatant. WT UPAB1 harboring pBAV::*invL*-*his_6_* was grown to early stationary phase, and WCL, sup, sup insoluble, and sup soluble fractions were isolated. Fractions were then probed for InvL (α-His_6_) and RNAP by immunoblotting. Numbers to the left indicate molecular weight in kDa. Two biological replicates were performed, yielding similar results, and a representative blot from one replicate is shown. Download FIG S2, TIF file, 0.9 MB. Copyright © 2022 Jackson-Litteken et al. 2022 Jackson-Litteken et al. https://creativecommons.org/licenses/by/4.0/ This content is distributed under the terms of the Creative Commons Attribution 4.0 International license.

### InvL binds to ECM components.

**FIG 5:**
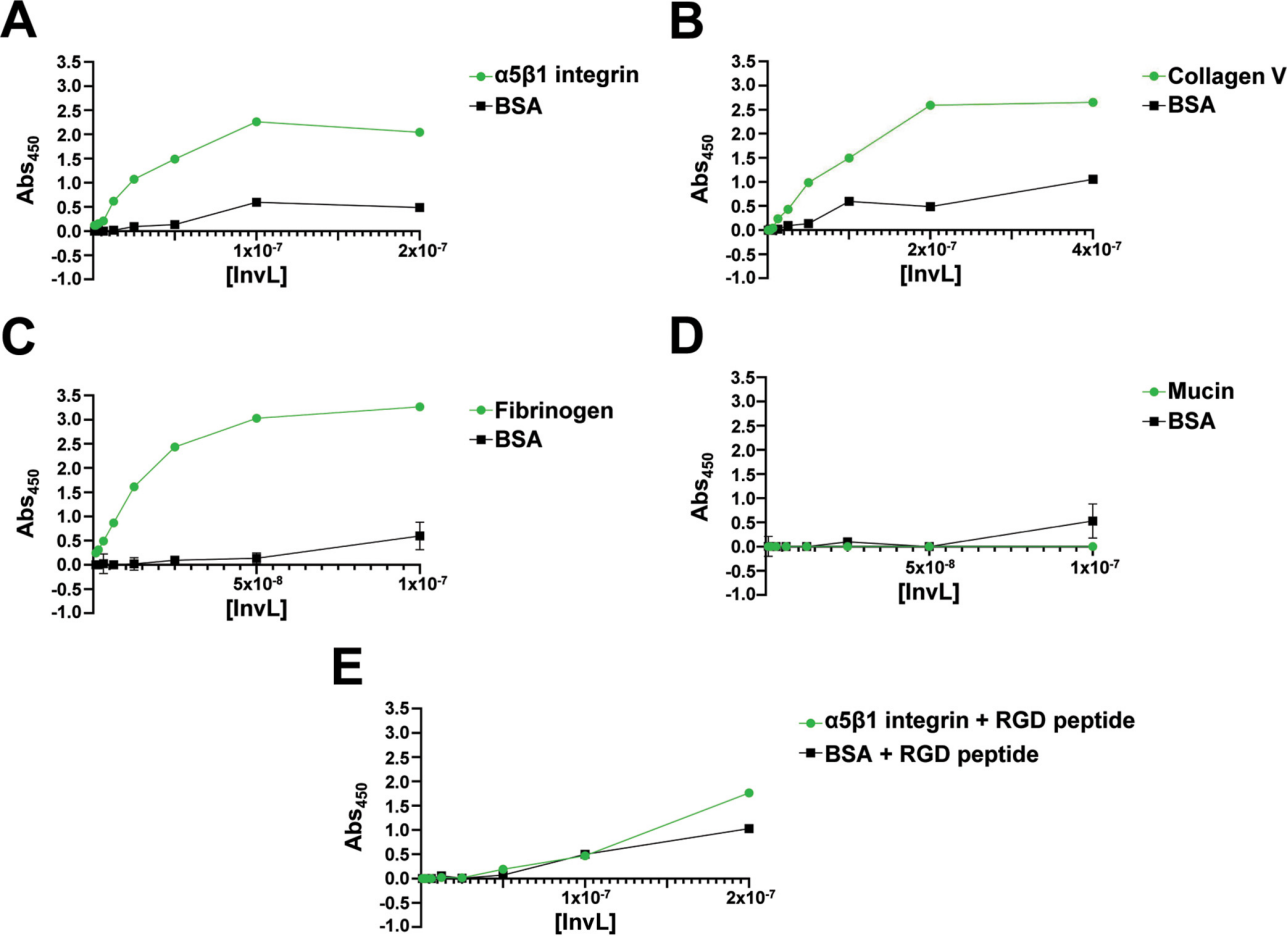
InvL binds to multiple ECM components. ELISAs were performed to assess binding of InvL to α5β1 integrin (A), collagen V (B), fibrinogen (C), mucin (D), or α5β1 integrin in the presence of RGD-containing peptide (E). BSA served as a negative binding control. Shown are the results from two biological replicates, and SEM is indicated by error bars.

We next examined whether InvL could bind to extracellular matrix (ECM) components. First, we used enzyme-linked immunosorbent assays (ELISAs) to investigate if InvL binds to α5β1 integrin (Fig. 5A) and collagen V (Fig. 5B). These molecules are the binding partners of InvA and an E. coli homolog, FdeC, respectively (39, 49, 50). We determined that InvL bound to α5β1 integrin and collagen V with dissociation constants (*K_d_*s) of 0.38 nM and 15 μM, respectively. On the contrary, InvL did not appreciably bind the negative control, bovine serum albumin (BSA), at the tested concentrations. Since UPAB1 colocalizes with fibrinogen deposited on the catheter during CAUTI, we assessed if InvL interacts with fibrinogen (10). We found that InvL bound to fibrinogen with the highest affinity of all ECM components tested (*K_d_* = 0.19 nM) (Fig. 5C). InvL, similar to InvA and FdeC, did not exhibit any significant binding to mucin, another common ECM component (Fig. 5D).

The interaction between InvA and β1 integrins is competitively inhibited by arginine-glycine-aspartate (RGD)-containing peptides, a motif required for the interaction of fibronectin with β1 integrin (51–53). RGD-containing peptides also abrogated binding of InvL to α5β1 integrin (Fig. 5E), indicating that InvL and InvA bind β1 integrins via similar mechanisms. Together, these results demonstrate that InvL binds α5β1 integrin, collagen V, and fibrinogen *in vitro*.

### InvL facilitates UPAB1 adhesion to bladder and kidney epithelial cells.

**FIG 6:**
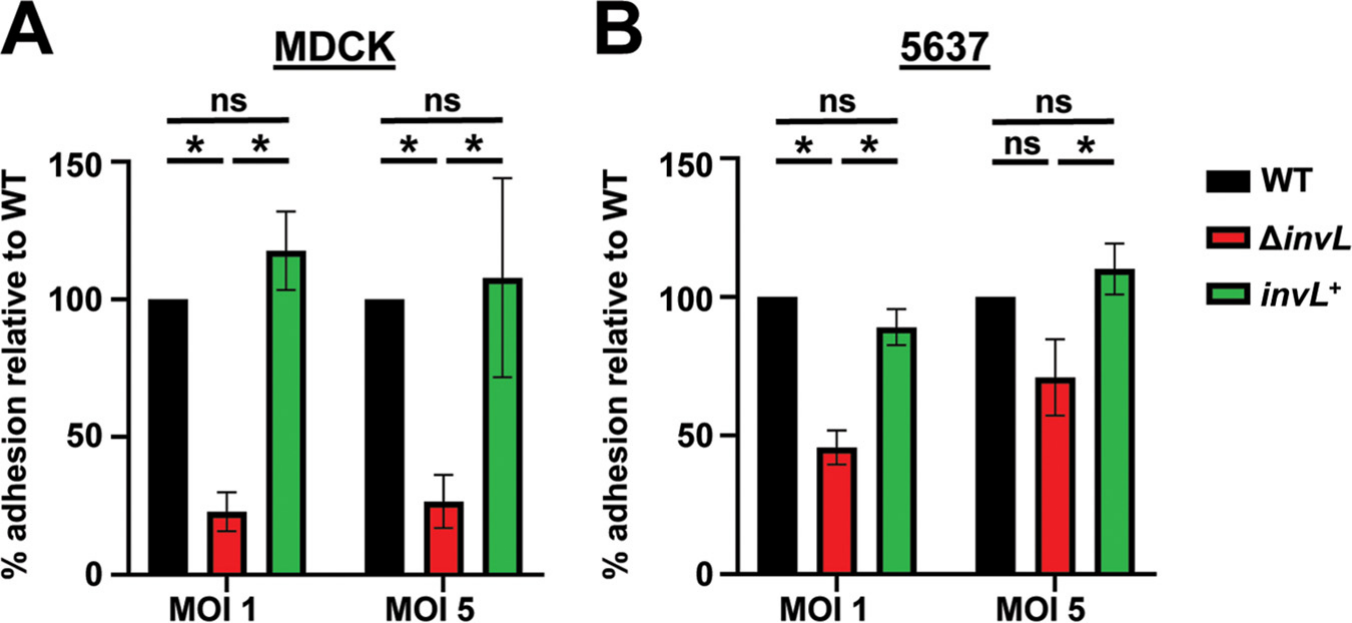
InvL facilitates adhesion to epithelial cells *in vitro*. UPAB1 WT, Δ*invL*, and *invL*^+^ strains were used in adhesion assays with MDCK (A) and 5637 (B) epithelial cells. The mean from six replicates is shown, and error bars represent the SEM. *, *P < *0.05; one-way ANOVA, Tukey’s test for multiple comparisons.

To examine if InvL plays a role in binding to epithelial cells relevant to the CAUTI model, we determined adhesion of UPAB1 WT, Δ*invL*, and *invL*^+^ strains to kidney (MDCK; Fig. 6A) and bladder (5637; Fig. 6B) cells. The Δ*invL* strain exhibited significantly decreased adhesion to both cell types relative to WT at a multiplicity of infection (MOI) of 1, and a similar trend toward decreased binding by the Δ*invL* strain was observed at an MOI of 5. This phenotype was reversed in the *invL*^+^ strain, confirming a role for InvL in urinary tract epithelial adhesion.

Because *Yersinia* InvA can mediate epithelial cell invasion, we performed antibiotic protection assays to assess if InvL performs a similar function (54–58). However, internalization was not observed in any of the cell lines tested, and immunofluorescence assays following epithelial cell infection confirmed UPAB1 is not invasive (Fig. S3). These results are not surprising given that few publications exist regarding Acinetobacter internalization in epithelial cells, and internalization results are highly variable depending on the strain and cell line tested (59–64). Altogether, our results demonstrate a role for InvL in binding to diverse epithelial cell lines, but a possible role in invasion in other strains or cells types cannot be ruled out.

10.1128/mbio.00258-22.7 FIG S3 A. baumannii is adhered extracellularly to 5637 epithelial cells. 5637 cells were infected with WT UPAB1 at an MOI of one, incubated for one h, and immunofluorescence microscopy was used to visualize intracellular versus extracellular bacteria. Nuclei are stained blue, actin is stained red, intracellular bacteria are stained green (rarely observed), and extracellular bacteria are double stained green and far-red. Two biological replicates were examined, and a representative image from one biological replicate is shown. The scale bar equals 20 μm. Download FIG S3, TIF file, 1.3 MB. Copyright © 2022 Jackson-Litteken et al. 2022 Jackson-Litteken et al. https://creativecommons.org/licenses/by/4.0/ This content is distributed under the terms of the Creative Commons Attribution 4.0 International license.

### InvL is required for full virulence in the CAUTI model.

**FIG 7:**
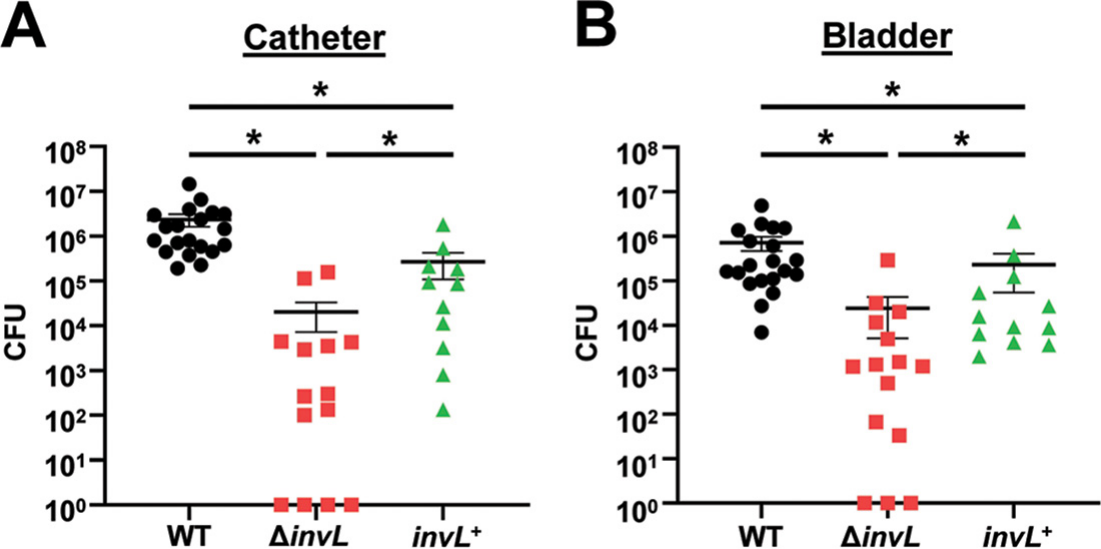
InvL is required for full virulence in a murine CAUTI model. Mice were implanted with a catheter followed by transurethral inoculation with UPAB1 WT, Δ*invL*, or *invL*^+^ strains. At 24 h postinfection, mice were sacrificed and bacterial burden on the catheter (A) and in the bladder (B) were quantified. Shown are results from at least three pooled experiments. Each data point represents an individual mouse, the horizontal line represents the mean, and the SEM is indicated by error bars. *, *P < *0.05; two-tailed Mann-Whitney *U* test.

Given the role of InvL as an adhesin, we tested if InvL contributes to Acinetobacter virulence in the murine CAUTI model. Mice were implanted with a catheter followed by transurethral inoculation with WT, Δ*invL*, or *invL*^+^ strains. At 24 h postinfection, mice were sacrificed, and bacteria adhered to the catheters (Fig. 7A) and bacterial burdens in the bladders (Fig. 7B) were quantified. The Δ*invL* mutant had a significant reduction in catheter binding, exhibiting approximately a 100-fold decrease relative to the WT strain, and this phenotype was partially reversed by genetic complementation. Δ*invL* also had a significant defect in bladder colonization relative to the WT strain, with more than 10-fold reduced CFU recovered. This phenotype was also partially complemented with the *invL*^+^ strain. The reason for partial complementation in the CAUTI model is unknown, but this result could be due to possible differential expression of *invL* in the complemented strain relative to WT bacteria. Nevertheless, these results indicate an important role for InvL in A. baumannii uropathogenesis.

### InvL is encoded by ACB complex strains and has high prevalence among international clone 2 isolates.

**FIG 8:**
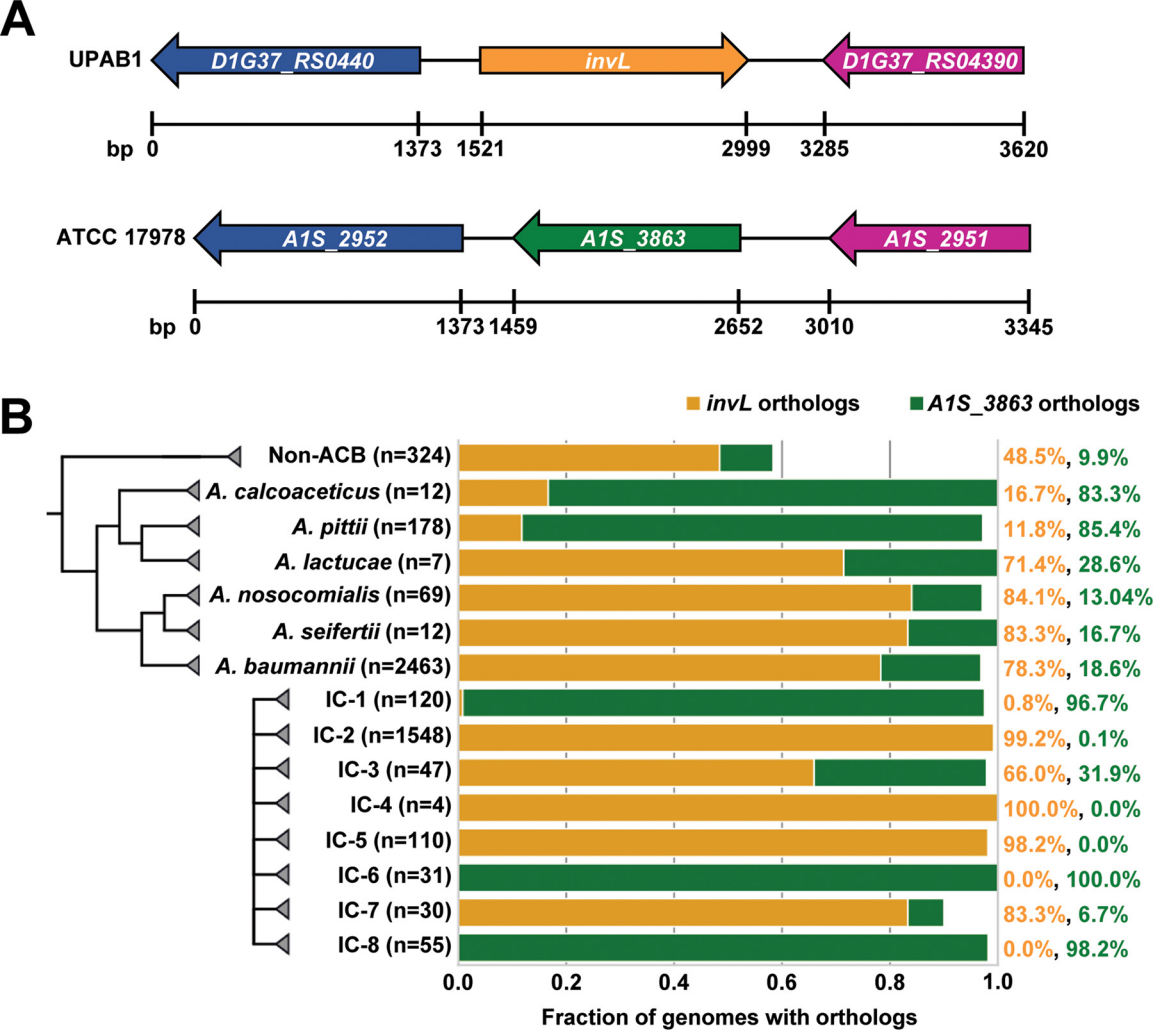
InvL is encoded by ACB complex strains and has high prevalence in IC-2 clones. (A) InvL is encoded at a common locus in ACB complex strains. Orthologs of InvL (top) and orthologs of A1S_3863 (bottom) are encoded at a common locus between genes encoding a dihydrolipoyl dehydrogenase (D1G37_RS0440 in UPAB1 and A1S_2952 in ATCC 17978; blue) and a TIGR01244 family phosphatase (D1G37_RS04390 in UPAB1 and A1S_2951 in ATCC 17978; purple). (B) Distribution of *invL* and *A1S_3863* in Acinetobacter. The fractions of genomes encoding InvL and A1S_3863 are reported by ACB species and international clone number. Percentage of genomes containing each ortholog by species and international clone number are indicated to the right.

As virulence factors could differ among isolates, we assessed the distribution of *invL* across the Acinetobacter genus. Specifically, we performed a targeted ortholog search for *invL* and adjacent genes in 3,052 available genomes from the National Center for Biotechnology Information (NCBI) RefSeq Database (65). In genomes that encode InvL, the corresponding gene is flanked by genes encoding a TIGR01244 family phosphatase (D1G37_04390 in UPAB1) and a dihydrolipoyl dehydrogenase (D1G37_RS04400 in UPAB1) (Fig. 8A). While the microsynteny of the two flanking genes is conserved throughout ACB complex, only 74% (2,029/2,728) of the isolates additionally harbor the *invL* gene. In 23% (641/2,728), a second cluster variant exists, where the *invL* gene is replaced by a gene encoding a hypothetical protein (A1S_3863 in ATCC 17978). The remainder of isolates (3%; 58/2728) have no/another intervening gene. The gene represented in the second cluster variant does not appear to be homologous to InvL. First, the two proteins reside on opposite strands, which interferes with genetic exchange via bacterial recombination. Second, at the amino acid sequence level, the two proteins are only 19% identical and 31% similar, and this is substantially driven by the shared presence of an N-terminal signal peptide. Third, the length and domain architecture differ between the proteins; InvL is about 100 amino acids longer, and it harbors an invasin/intimin cell-adhesion domain (IPR008964) that is missing in the other protein. Outside the ACB complex, the gene cluster is represented in only 216/324 (67%) of the isolates. Of these, again the majority encode InvL (157/216; 72%). The second cluster variant is present in 32/216 (15%) of the isolates, and the remaining 13% have either no or another intervening gene. Of note, InvL distribution correlates with international clone (IC) number, as nearly all strains belonging to IC-2, IC-4, and IC-5 groups encode InvL (Fig. 8B). Alternatively, strains belonging to IC-1, IC-6, and IC-8 predominantly encode the second cluster variant. As IC-2 clones are among the most commonly isolated in clinical settings, the prevalence of InvL in these strains is intriguing (1–3, 66, 67). However, the fact that other international clones that are associated with human infection do not predominantly encode InvL (e.g., IC-1) implies that some other protein(s) compensates for the absence of this adhesin. In sum, we conclude that InvL is prevalent in the ACB complex and is found in nearly all IC-2 strains, which are relevant to human disease.

## DISCUSSION

Acinetobacter has emerged as a pathogen of significant clinical importance due to the increasing frequency of multidrug-resistant strains identified (6, 8, 9). Approximately 20% of isolates are derived from the urinary tract, highlighting the uropathogenic potential of A. baumannii (10, 11). Mutation of the T2SS in the uropathogenic strain UPAB1 resulted in decreased catheter-binding and lower burden in the bladder, indicative of a key role during urinary tract virulence. Through a proteomic analysis, we identified a surface-exposed T2SS effector that belongs to the intimin-invasin family, referred to here as InvL. We found that recombinant InvL binds to host ECM components and that the UPAB1 Δ*invL* mutant strain has a significant defect in binding to bladder and kidney epithelial cells. Accordingly, the Δ*invL* mutant was attenuated in the CAUTI model. In all, our study demonstrates the importance of the T2SS for Acinetobacter uropathogenesis and describes a novel T2SS effector, InvL, as a previously unrecognized adhesin and virulence factor that is encoded by most clinical isolates.

Interestingly, although protein modeling revealed structural similarity of InvL to *Yersinia* InvA, InvL is unique compared to other invasin homologs. First, while canonical invasin homologs are T5SSs, outer membrane localization of InvL is dependent on the T2SS. To our knowledge, this is the first report of an intimin-invasin family T2SS effector. Second, unlike other InvA homologs, which are anchored to the outer membrane via a large N-terminal β-barrel domain, InvL is predicted to be a lipoprotein based on the presence of a lipoprotein secretion signal (36, 41). While the T2SS is known to secrete soluble effectors into the extracellular milieu, it has also been reported to be essential for surface localization of some lipoproteins, similar to what is reported here for InvL (68). One of the best-characterized examples is the T2SS-dependent surface localization of the lipoprotein pullulanase (PulA), a starch-debranching enzyme of Klebsiella oxytoca (69–71). PulA, like many other lipoproteins, is translocated from the cytoplasm to the periplasm via the Sec pathway, where processing and acylation occur to attach the protein to the periplasmic leaflet of the inner membrane (70, 72). Whereas most outer membrane lipoproteins of Gram-negative bacteria are transported from the inner membrane to the outer membrane via the localization of lipoproteins (Lol) pathway, PulA uses the T2SS machinery (69–71). The mechanism by which the T2SS can recognize its lipoprotein effectors and transport lipidated proteins across the periplasm and outer membrane remains elusive. However, this report of InvL adds to the growing list of known T2SS effectors which are surface exposed lipoproteins (68). As several of these proteins are involved in pathogenesis and key metabolic functions (e.g., SslE of E. coli and MtrC and OmcA of Shewanella oneidensis), a better understanding of the mechanism of T2SS-dependent lipoprotein transport is needed (68, 73–78).

Interestingly, in addition to being a surface-exposed protein, we found that InvL is secreted into the supernatant. This is somewhat surprising, as this has not been reported with other members of the intimin-invasin family to our knowledge. Given that InvL localized to the insoluble fraction of the supernatant, it is possible that InvL is found in OMVs. Notably, this release of a T2SS-dependent surface-associated lipoprotein into the media is reminiscent of PulA (79, 80). However, it is unclear if PulA is associated with OMVs, and we cannot discount the possibility that the identification of PulA and InvL in the supernatant is due to their localization in bacterial lysis byproducts (68). Regardless, it is tempting to speculate that secreted InvL could serve an alternative function (e.g., modulation of host responses). However, whether InvL serves any roles other than being an adhesin is outside the scope of this manuscript and will be the subject of future work.

Similar to other members of the intimin-invasin protein family, InvL can directly bind to ECM components and facilitate cell adhesion. Specifically, InvL binds with high affinity to α5β1 integrin, collagen V, and fibrinogen. α5β1 integrin and collagen V are also bound by the intimin-invasin family proteins InvA and FdeC of *Yersinia* and E. coli, respectively (39, 50). Interestingly though, InvA and FdeC appear to bind to a single defined ECM component, indicating that InvL has a broader specificity. While domains/residues required for the FdeC-collagen interaction have not been defined, the interaction between InvA and β1 integrins has been extensively studied (49, 81–85). Binding of InvA to β1 integrins specifically requires the C-terminal lectin-like domain, which is present in InvL and absent from FdeC (50, 83, 85). It is tempting to speculate that the predicted InvL lectin-like domain is involved in binding to α5β1 integrin and that characteristics of Ig-like domains are responsible for recognition of collagen V. However, it should be noted that residues known to be important for the interaction between InvA and β1 integrins do not appear to be conserved in InvL (81–84). This is despite the observation that the interactions of InvL and InvA with α5β1 integrin can be similarly inhibited by RGD peptide (51–53). The low sequence identity between InvL and invasin homologs makes it difficult to accurately predict key regions of the protein required for recognition of ECM components. The structural characterization of InvL will, in the future, provide mechanistic insights into the interaction between InvL and the ECM.

Our characterization of InvL adds to the growing list of known Acinetobacter adhesins that facilitate binding to epithelial cells, which includes type IV pili, the Ata autotransporter, the FhaB/FhaC two-partner secretion system, and biofilm-associated protein (Bap) (86–96). ECM components bound by type IV pili and Bap are not known. However, Ata has been shown to bind with high affinity to collagen (types I, III, IV, and V), laminin, and glycosylated fibronectin, and FhaBC reportedly binds to fibronectin (88, 91, 92, 95). While the collagen-binding role of InvL may be redundant with that of Ata, InvL is the first published Acinetobacter adhesin to bind directly to α5β1 integrin or fibrinogen. Additionally, InvL is now the first known adhesin to be important for A. baumannii UTIs. Future work will determine the roles of these known adhesins in the multiple niches colonized by this bacterium.

While we have established that InvL is a virulence factor involved in Acinetobacter uropathogenesis, the mechanism(s) by which InvL participates in CAUTI is unknown. The fact that we identified fibrinogen as the highest-affinity binding partner for InvL is particularly intriguing with respect to CAUTI. Tissue damage from catheterization results in an inflammatory response that leads to increased levels of fibrinogen in the bladder that ultimately coats the catheter (97, 98). We recently showed that Acinetobacter, similar to other uropathogens such as Enterococcus faecalis and Staphylococcus aureus, colocalizes with fibrinogen on the catheter (10, 99, 100). This interaction is dependent on the virulence factors EbpA and ClfB in E. faecalis and S. aureus, respectively (98, 99). It is tempting to speculate that InvL serves a similar function with respect to Acinetobacter catheter binding. However, future work is required to examine if InvL mediates catheter binding via fibrinogen or if the ability of InvL to associate with other ECM components is responsible for its role in the CAUTI model. Additionally, future work is required to determine if InvL similarly serves as a virulence factor in other infection models (e.g., pneumonia and bacteremia). Ultimately, a better understanding of A. baumannii virulence factors such as InvL may aid in the development of novel therapeutics to combat infection by these increasingly multidrug-resistant bacteria.

## MATERIALS AND METHODS

### Bacterial plasmids, strains, and growth conditions.

Plasmids and strains used in this study are detailed in Table S2 in the supplemental material. Cultures were grown at 37°C using Lennox broth/agar unless otherwise noted. The following antibiotic concentrations were used when appropriate: 10 μg/mL chloramphenicol, 100 μg/mL ampicillin, 30 μg/mL apramycin, or 50 μg/mL zeocin for E. coli and 10 μg/mL chloramphenicol, 50 μg/mL apramycin, 50 μg/mL zeocin, or 300 μg/mL hygromycin B for A. baumannii.

10.1128/mbio.00258-22.3 TABLE S2 Plasmids and strains used in this study. Download Table S2, DOCX file, 0.06 MB. Copyright © 2022 Jackson-Litteken et al. 2022 Jackson-Litteken et al. https://creativecommons.org/licenses/by/4.0/ This content is distributed under the terms of the Creative Commons Attribution 4.0 International license.

### Generation of constructs used in this study.

Plasmids and primers used in this study are detailed in Tables S2 and S3, respectively. Detailed information for construction of plasmids and strains used in the study is provided in the supplemental material.

### Murine infection experiments.

Animal experiments were approved by the Washington University Institutional Animal Care and Use Committee. The murine CAUTI model was performed as previously described (10). Briefly, 6- to 8-week-old female C57BL/6 mice (Charles River Laboratories, Wilmington, MA) anesthetized with 4% isoflurane were implanted transurethrally with a 4- to 5-mm piece of silicon tubing (catheter). Once-passaged statically grown bacterial strains were washed twice and resuspended in phosphate-buffered saline (PBS). Mice were then transurethrally inoculated with 50 μL of the bacterial suspension containing 2 × 10^8^ CFU. At 24 h postinfection, mice were sacrificed, and bacterial load on catheters and in bladders were quantified by serial dilution plating.

### Proteomic analysis of the T2SS-dependent secretome.

Bacterial culture conditions, secreted protein enrichment, digestion of secretome samples, and liquid chromatography-mass spectrometry analysis of secretome samples were as previously described (101). Details are provided in the supplemental material.

### Immunoblot analyses.

Bacterial whole-cell lysate preparation and trichloroacetic acid precipitation of supernatant fractions from mid-exponential cultures was performed as previously described (24, 104). Indicated fractions were separated by SDS-PAGE, transferred to a nitrocellulose membrane, and proteins of interest were probed using polyclonal rabbit anti-His_6_ (1:2,000; Invitrogen, Waltham, MA) and monoclonal mouse anti-RNAP (1:3,500; BioLegend, San Diego, CA). IRDye-conjugated anti-mouse IgG and anti-rabbit IgG were used as secondary antibodies (1:5,000 for each; LI-COR Biosciences, Lincoln, NE), and blots were visualized with the Odyssey CLx imaging system (LI-COR Biosciences).

### Proteinase K accessibility assays.

Proteinase K accessibility assays were performed as previously described, with some modification (105). Stationary-phase cultures were pelleted and resuspended in PBS supplemented with 5 mM MgCl_2_ to an optical density at 600 nm (OD_600_) of 2.5. Bacterial suspensions were treated with 200 μg/mL proteinase K (GoldBio, Olivette, MO) or sham treated with an equal volume of deionized water in the presence or absence of 2% Triton X-100 (Sigma-Aldrich, St. Louis, MO) at 37°C for 15 min. Digestion was terminated by addition of 1 mM phenylmethylsulfonyl fluoride (Amresco LLC, Salon, OH), Laemmli buffer was added to a concentration of 1×, and samples were boiled for 10 min. 10-μL samples of these preparations were separated by SDS-PAGE, and immunoblotting was performed as described above.

### Expression and purification of recombinant InvL.

To generate recombinant protein for antibody generation, full-length InvL with a C-terminal His_10_ tag was expressed from pET-22b(+)::*invL*-*his_10_* in Rosetta-gami 2(DE3) cells (Novagen, Madison, WI) using ZYM-5052 autoinducible medium for 72 h at 20°C (106). Cells were then lysed using a CF1 cell disrupter (Constant Systems Ltd., Daventry, UK). Following cell lysis, recombinant InvL was purified from the insoluble fraction by Ni-nitrilotriacetic acid affinity chromatography as previously described (107). Purified protein was then used by Antibody Research Corporation (St. Peters, MO) to generate polyclonal rabbit antibody.

To generate soluble recombinant InvL for ELISA (described below), InvL lacking the N-terminal signal sequence (as determined by SignalP 5.0) was expressed with a C-terminal His_10_ tag from pET-22b(+)::*invL-his_10_*(-SS) in Rosetta-gami 2(DE3) cells by addition of 1 mM isopropyl-β-d-thiogalactopyranoside for 3 h at 37°C (41). Cells were then lysed as described above, and recombinant InvL was purified from the soluble fraction using the procedure previously described with the exception that buffers were not supplemented with 0.3% *N*-lauryl-sarcosine (107).

### ELISAs.

ELISAs were based on a previously described protocol (50). Detailed protocol information can be found in the supplemental material.

### Eukaryotic cell adhesion assays.

Cell adhesion assays were performed with MDCK and 5637 cells cultured in Dulbecco's Modified Eagle Medium (DMEM) and Roswell Park Memorial Institute 1640 (RPMI-1640) (Gibco, Dublin, Ireland) supplemented with 10% heat-inactivated fetal bovine serum, respectively. 10^5^ MDCK and 3 × 10^5^ 5637 cells (amounts determined to form confluent monolayers) were plated in 48-well Nunclon Delta cell culture–treated surface plates (ThermoFisher Scientific) overnight at 37°C with 5% CO_2_. Stationary-phase A. baumannii strains were pelleted at 5,432 × *g* for 5 min and resuspended in PBS. Following one more spin, bacteria were resuspended in the appropriate cell culture media, and 500 μL of suspension at the indicated MOI was added to the appropriate wells. Plates were then centrifuged at 200 × *g* and incubated at 37°C with 5% CO_2_ for 1 h to allow adhesion. Wells were subsequently washed three times with 500 μL PBS, cells were resuspended in 0.05% Triton X-100 in PBS, and serial dilution plating was performed to quantify bacteria. Concurrently, to quantify intracellular bacteria after the 1 h of incubation and washing, the appropriate cell culture medium supplemented with 50 μg/mL colistin was added for 1 h. After washing in PBS, cells were resuspended in 0.05% Triton X-100 in PBS, and serial dilution plating was performed to quantify bacteria.

### Bioinformatic analysis of InvL distribution.

Ortholog searches were performed across a database of 3,052 Acinetobacter genomes obtained from NCBI RefSeq as previously described (65). Briefly, the collection of orthologous groups that resulted from an all versus all ortholog search using OMA was screened to associate the group that harbors a particular protein sequence of interest (108). The sequences of the associated group collectively served to train a model for the targeted ortholog search across the full database of genomes. The proteins of interest comprise the translated sequences of D1G37_RS04395 (InvL), D1G37_RS0440, D1G37_RS04390, and A1S_3863. Since the subset of genomes used by Djahanschiri et al. did not include the strain UPAB1, the orthologs identified in the strain MDR-TJ via a reciprocal best blast hit approach were used to associate orthologous groups (65). The resulting presence/absence matrix together with the genus’ phylogeny served as the input for Vicinator v0.32 (https://github.com/BIONF/Vicinator) to trace the microsynteny of the *invL* locus based on MDR-TJ and ATCC 17978 as the reference genomes.

### Data availability.

The MS data and search results have been deposited into the PRIDE ProteomeXchange Consortium repository and can be accessed using the accession numbers PXD030460 and PXD030491 (102, 103). All other data are provided within the text or supplemental material.

### Statistical methods.

All statistical analyses were performed using GraphPad Prism version 9.

10.1128/mbio.00258-22.1 TEXT S1 Supplemental materials and methods. Download Text S1, DOCX file, 0.1 MB. Copyright © 2022 Jackson-Litteken et al. 2022 Jackson-Litteken et al. https://creativecommons.org/licenses/by/4.0/ This content is distributed under the terms of the Creative Commons Attribution 4.0 International license.

10.1128/mbio.00258-22.4 TABLE S3 Primers used in this study. Download Table S3, DOCX file, 0.08 MB. Copyright © 2022 Jackson-Litteken et al. 2022 Jackson-Litteken et al. https://creativecommons.org/licenses/by/4.0/ This content is distributed under the terms of the Creative Commons Attribution 4.0 International license.
