# Supplementary material for: InvL, an Invasin-Like Adhesin, Is a Type II Secretion System Substrate Required for Acinetobacter baumannii Uropathogenesis
Source: mBio. 2022 May 31;13(3):e00258-22. doi: 10.1128/mbio.00258-22 (PMC9245377; doi:10.1128/mbio.00258-22)
Supplement: TABLE S1 [file mbio.00258-22-s0006.docx]

**Table S1. Putative T2SS-dependent secretome of UPAB1**

| Average fold-change (WT vs. Δ*gspD*) | Locus Tag | Annotation | Signal Peptide^a^ |
| --- | --- | --- | --- |
| 12.34 | D1G37_RS10930 | 5'-methylthioadenosine/S-adenosylhomocysteine nucleosidase | Lipoprotein signal peptide (Sec/SPII) |
| 11.80 | D1G37_RS04395 | hypothetical protein | Lipoprotein signal peptide (Sec/SPII) |
| 11.47 | D1G37_RS18220 | metalloendopeptidase CpaA | Signal peptide (Sec/SPI) |
| 9.27 | D1G37_RS02935 | glycerophosphodiester phosphodiesterase | Lipoprotein signal peptide (Sec/SPII) |
| 9.26 | D1G37_RS16055 | histidine-type phosphatase | Lipoprotein signal peptide (Sec/SPII) |
| 8.95 | D1G37_RS01360 | alpha/beta fold hydrolase | Lipoprotein signal peptide (Sec/SPII) |
| 8.71 | D1G37_RS11575 | gamma-glutamyltransferase | Lipoprotein signal peptide (Sec/SPII) |
| 8.66 | D1G37_RS10450 | hypothetical protein | Lipoprotein signal peptide (Sec/SPII) |
| 7.95 | D1G37_RS16990 | 5'/3'-nucleotidase SurE | Signal peptide (Sec/SPI) |
| 7.82 | D1G37_RS16605 | bifunctional metallophosphatase/5'-nucleotidase | Lipoprotein signal peptide (Sec/SPII) |
| 6.83 | D1G37_RS12710 | tannase/feruloyl esterase family alpha/beta hydrolase | Lipoprotein signal peptide (Sec/SPII) |
| 6.79 | D1G37_RS08070 | esterase-like activity of phytase family protein | Lipoprotein signal peptide (Sec/SPII) |
| 5.85 | D1G37_RS00090 | hypothetical protein | Lipoprotein signal peptide (Sec/SPII) |
| 5.76 | D1G37_RS16985 | hypothetical protein | Lipoprotein signal peptide (Sec/SPII) |
| 5.14 | D1G37_RS11690 | transferrin-binding protein-like solute binding protein | Signal peptide (Sec/SPI) |
| 4.97 | D1G37_RS00130 | chorismate mutase | Signal peptide (Sec/SPI) |
| 4.81 | D1G37_RS16720 | type 1 fimbrial protein | Signal peptide (Sec/SPI) |
| 4.48 | D1G37_RS01565 | hypothetical protein | Lipoprotein signal peptide (Sec/SPII) |
| 4.38 | D1G37_RS17565 | hypothetical protein | Lipoprotein signal peptide (Sec/SPII) |
| 3.83 | D1G37_RS11685 | TonB-dependent receptor | Signal peptide (Sec/SPI) |
| 3.73 | D1G37_RS02600 | CSLREA domain-containing protein | Signal peptide (Sec/SPI) |
| 3.71 | D1G37_RS18465 | hypothetical protein | Signal peptide (Sec/SPI) |
| 3.63 | D1G37_RS17085 | cupin domain-containing protein | Signal peptide (Sec/SPI) |
| 3.46 | D1G37_RS12010 | arylsulfatase | Lipoprotein signal peptide (Sec/SPII) |
| 3.13 | D1G37_RS02915 | lytic transglycosylase domain-containing protein | Signal peptide (Sec/SPI) |
| 3.12 | D1G37_RS05565 | hypothetical protein | Signal peptide (Sec/SPI) |
| 2.98 | D1G37_RS06350 | transporter | Signal peptide (Sec/SPI) |
| 2.62 | D1G37_RS00315 | hypothetical protein | Lipoprotein signal peptide (Sec/SPII) |
| 2.56 | D1G37_RS02605 | rhombotarget A | Signal peptide (Sec/SPI) |

^a^Signal peptides identified by SignalP 5.0 (1)

**REFERENCES**

1. Petersen TN, Brunak S, von Heijne G, Nielsen H. 2011. SignalP 4.0: discriminating signal peptides from transmembrane regions. Nat Methods 8:785–786.
