# Supplementary figures and images for: InvL, an Invasin-Like Adhesin, Is a Type II Secretion System Substrate Required for Acinetobacter baumannii Uropathogenesis
Source: mBio. 2022 May 31;13(3):e00258-22. doi: 10.1128/mbio.00258-22 (PMC9245377; doi:10.1128/mbio.00258-22)

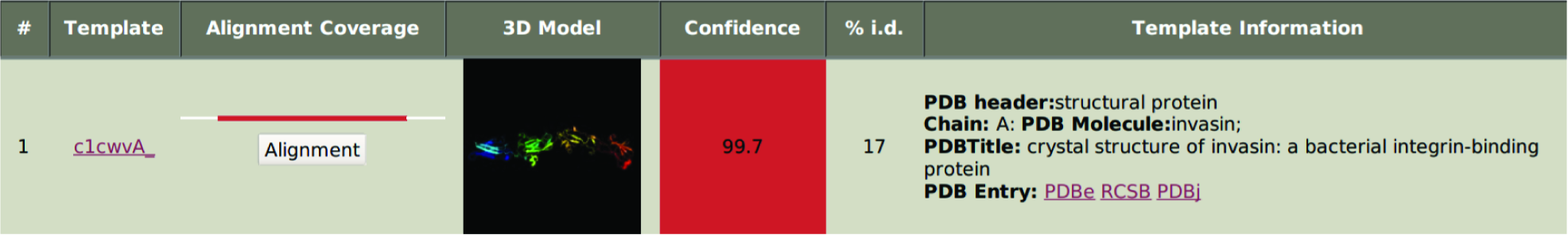

Supplement: FIG S1 [file mbio.00258-22-s0001.tif]

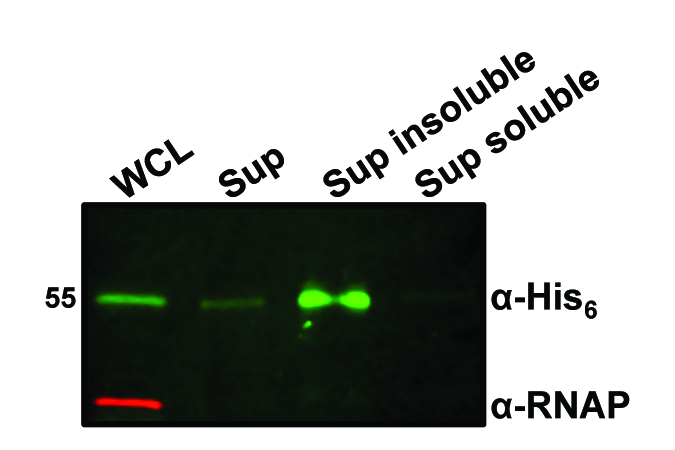

Supplement: FIG S2 [file mbio.00258-22-s0002.tif]

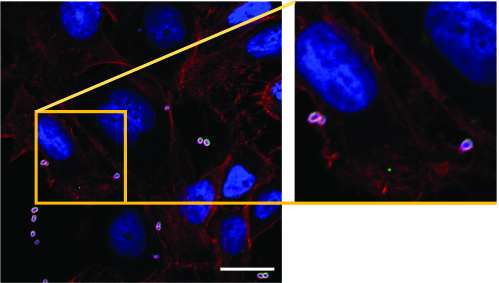

Supplement: FIG S3 [file mbio.00258-22-s0003.tif]
