# Supplementary material for: InvL, an Invasin-Like Adhesin, Is a Type II Secretion System Substrate Required for Acinetobacter baumannii Uropathogenesis
Source: mBio. 2022 May 31;13(3):e00258-22. doi: 10.1128/mbio.00258-22 (PMC9245377; doi:10.1128/mbio.00258-22)
Supplement: TABLE S2 [file mbio.00258-22-s0007.docx]

**Table S2. Plasmids and strains used in this study.**

| **Plasmid or Strain** | **Description**^a^ | **Source** |
| --- | --- | --- |
| Plasmids |  |  |
| pKD4-Zeo | Source for zeocin cassette for mutant generation, Zeo^r^ | (1, 2) |
| pUC18T-miniTn7T-Zeo | mTn7 complementation vector, Zeo^r^ | (3) |
| pUC18T-miniTn7T-Zeo::*gspD* | Δ*gspD* complementation construct, Zeo^r^ | This study |
| pUC18T-miniTn7T-Zeo::*fdeC*-*his_6_* | Δ*invL* complementation construct, Zeo^r^ | This study |
| pBAV1K-T5-gfp | Vector used for generating pBAV-Apr; Kan^r^ | (4) |
| pUC18T-miniTn7T-Apr | Vector harboring apramycin resistance cassette used for generation of pBAV::*invL*-*his_6_*, Apr^r^ | (3) |
| pBAV-Apr::*gfp* | pBAV-Apr harboring a *gfp* cassette, Apr^r^ | This study |
| pBAV-Apr::*invL*-*his_6­_* | pBAV-Apr expressing *invL* with a His_6_ tag from its native promoter; Apr^r^ | This study |
| pET-22b(+) | Expression vector; Amp^r^ | Novagen |
| pET-22b(+)::*invL*-*his_10_* | pET-22b(+) expressing full-length *invL* with a C-terminal His_10_ tag; Amp^r^ | This study |
| pET-22b(+)::*invL*-*his_10_*(-SS) | pET-22b(+) expressing *invL* without the signal sequence and with a C-terminal His_10_ tag; Amp^r^ | This study |
| Strains |  |  |
| *E. coli* |  |  |
| Stellar | *mrr-hsdRMS-mcrBC* and *mcrA* | TaKaRa |
| Rosetta-Gami 2 (DE3) | *relA1 lac* (DE3) F′[*proA+ B+ lacIqZΔM15*::*Tn10*] pRARE*;* Cam^r^ | Novagen |
| *A. baumannii* |  |  |
| UPAB1 | WT | (5) |
| UPAB1 Δ*gspD* | UPAB1 *gspD* mutant | This study |
| UPAB1 *gspD*+ | UPAB1 *gspD* mutant complemented | This study |
| UPAB1 Δ*invL* | UPAB1 *invL* mutant | This study |
| UPAB1 *invL*+ | UPAB1 *invL* mutant complemented | This study |

^a^Zeo, zeocin; Amp, ampicillin; Apr, apramycin; Cam, chloramphenicol

**REFERENCES**

1. Datsenko KA, Wanner BL. 2000. One-step inactivation of chromosomal genes in *Escherichia coli* K-12 using PCR products. Proc Natl Acad Sci U S A 97:6640–6645.

2. Le NH, Peters K, Espaillat A, Sheldon JR, Gray J, Venanzio G di, Lopez J, Djahanschiri B, Mueller EA, Hennon SW, Levin PA, Ebersberger I, Skaar EP, Cava F, Vollmer W, Feldman MF. 2020. Peptidoglycan editing provides immunity to *Acinetobacter baumannii* during bacterial warfare. Sci Adv 6.

3. Ducas-Mowchun K, de Silva PM, Crisostomo L, Fernando DM, Chao TC, Pelka P, Schweizer HP, Kumar A. 2019. Next Generation of Tn 7-Based Single-Copy Insertion Elements for Use in Multi- and Pan-Drug-Resistant Strains of *Acinetobacter baumannii*. Appl Environ Microbiol 85.

4. Bryksin A v., Matsumura I. 2010. Rational design of a plasmid origin that replicates efficiently in both gram-positive and gram-negative bacteria. PLoS One 5.

5. di Venanzio G, Flores-Mireles AL, Calix JJ, Haurat MF, Scott NE, Palmer LD, Potter RF, Hibbing ME, Friedman L, Wang B, Dantas G, Skaar EP, Hultgren SJ, Feldman MF. 2019. Urinary tract colonization is enhanced by a plasmid that regulates uropathogenic *Acinetobacter baumannii* chromosomal genes. Nat Commun 10.
