# Supplementary material for: InvL, an Invasin-Like Adhesin, Is a Type II Secretion System Substrate Required for Acinetobacter baumannii Uropathogenesis
Source: mBio. 2022 May 31;13(3):e00258-22. doi: 10.1128/mbio.00258-22 (PMC9245377; doi:10.1128/mbio.00258-22)
Supplement: TEXT S1 [file mbio.00258-22-s0004.docx]

**SUPPLEMENTAL MATERIALS AND METHODS**

**Generation of constructs/strains used in this study.** Assembly of DNA fragments was performed using the In-Fusion HD EcoDry Cloning Kit (TaKaRa Bio, Mountain View, CA). UPAB1 mutants were made as previously described (1). Briefly, regions upstream (Δ*gspD* primers: 5’ F1 gspDKO and 3’ F1 gspDKO; Δ*invL* primers: AB FdeC Fwd and AB FdeC P1 Rev) and downstream (Δ*gspD* primers: 5’ F2 gspDKO and 3’ F2 gspDKO; Δ*invL* primers: CD FdeC P2 Fwd and CD FdeC Rev) of genes to be deleted were amplified from UPAB1 genomic DNA (gDNA) and fused together with an FRT site-flanked zeocin resistance cassette between (primers: P1 and P2) amplified from a variant of pKD4 by overlap extension PCR (2, 3). The PCR product was electroporated into WT UPAB1 harboring pAT04, which encodes an inducible copy of RecAB recombinase (1). The mutation was subsequently selected for with zeocin treatment. To remove the zeocin resistance cassette, mutants were transformed with the plasmid pAT03, which encodes an inducible copy of FLP recombinase (1). Clean mutants were then confirmed by PCR analyses. Genetic complementation was achieved using a mini-Tn7 system as previously described (4, 5). Briefly, pUC18T-miniTn7-Zeo was amplified (Δ*gspD* primers: 5’ pUC for gspD and 3’ pUC for gspD; Δ*invL* primers: His-pTn7 linear Fwd and Tn7 linear Rev) and fused together with the gene of interest along with the upstream putative promoter region (Δ*gspD* primers: 5’ gspD Comp and 3’ gspD Comp; Δ*invL* primers: prom FdeC Fwd and His-FdeC- Rev) (2, 6). The copy of the complemented gene and the zeocin resistance cassette were then introduced into the chromosome using a four-parental conjugation technique (4–7). Complementation was confirmed by PCR analyses.

A vector derived from pBAV1K-T5-gfp was used to express *invL* with a His_6_ tag for detection in bacterial lysates/supernatants (8). To generate pBAV-Apr::*gfp*, the kanamycin resistance cassette was replaced with an apramycin resistance cassette by amplifying pBAV1K-T5-gfp without the kanamycin resistance cassette (primers: 5’ pBAV linear marker swap and 3’ pBAV linear marker swap) and the apramycin cassette from pUC18T-miniTn7T-Apr (primers: 5’ Apr for pBAV and 3’ Apr for pBAV v2) and assembling the two fragments together (6). The *gfp* cassette in pBAV-Apr::*gfp* was then replaced with *invL* with a C-terminal His_6_ tag and its native promoter region by amplifying the vector backbone from pBAV-Apr::*gfp* [primers: N-term HisTag Fwd (for pBAV/fdeC) and 3’ pBAV for UPAB1 fdeC natprom] and *invL* from UPAB1 gDNA [primers: 5’ UPAB1 fdeC natprom for pBAV and fdeC REV (for pBAV)] and assembling the products. This final construct was designated pBAV-Apr::*invL*-*his_6­_*.

To generate pET-22b(+)-derivative vectors for protein expression, first full-length *invL* (primers: fdeC NdeI Fwd and fdeC His noEND Rev) and the pET-22b(+) vector backbone (primers: pET-22b linear Fwd and pET-22b linear Rev) were amplified and assembled, generating pET-22b(+)::*invL*. This plasmid was then used as the template to amplify *invL* with a His_10_ tag (primers: fdeC NdeI Fwd v2 and FdeC 10His Rev) and the pET-22b(+) backbone (primers: pET-22b linear Fwd and pET-22b linear Rev), and these segments were assembled, generating pET-22b(+)::*invL*-*his_10_*. pET-22b(+)::*invL*-*his_10_* was subsequently used to amplify the *invL* without the signal sequence (primers: 5’ fdeC-SS for pET-22B and FdeC 10His Rev) and the pET22b(+) backbone (primers: pET-22b linear Fwd and pET-22b linear Rev v2). These segments were then assembled, generating pET-22b(+)::*invL*-*his_10_*(-SS).

**ELISAs.** ELISAs were based on a previously described protocol (9). Costar 96-well high binding, flat bottom plates (Corning Inc, Corning, NY) were coated with the 1 μg of recombinant human (CHO cell-derived) α5β1 integrin (R&D Systems, Minneapolis, MN), collagen V from human placenta (MilliporeSigma, Burlington, MA), fibrinogen from human plasma (Sigma-Aldrich), mucin from bovine submaxillary glands (Sigma-Aldrich), or BSA (ThermoFisher Scientific, Waltham, MA) in 200 μl PBS overnight at 4°C. Subsequently, wells were washed with 200 μl ELISA wash buffer (0.5X PBS; 0.05% Tween) three times with gentle rocking at room temperature and then incubated stationary at 37°C for 2 h with 250 μl ELISA blocking buffer [ELISA wash buffer supplemented with 2.7% polyvinylpyrrolidone (PVP)]. After blocking and washing, the 96-well plate was incubated overnight in 200 μl PBS at 4°C. Wells were then washed three times followed by addition of recombinant InvL (see above) in sequential two-fold serial dilutions in PBS at the indicated concentrations. For the competition ELISA, RGD-containing peptide (H-Gly-Arg-Gly-Asp-Ser-Pro-OH; MilliporeSigma) was spiked into InvL serial dilutions at a concentration of 20X the K_d_. Plates were then incubated stationary at 37°C for 2 h, followed by washing. 200 μl ELISA wash buffer supplemented with 0.27% PVP and rabbit polyclonal antibody to InvL (see above) at a ratio of 1:5000 was added to the wells, and the plate was incubated stationary at 37°C for 1.5 h. Subsequently, wells were washed again, 200 μl ELISA wash buffer supplemented with 0.27% PVP and 1:10000 goat anti-rabbit antibody conjugated to horse radish peroxidase (Bio-Rad Laboratories, Hercules, CA) was added, and the plates were incubated stationary for 1.5 h at 37°C. The wells were then washed, and 100 μl aSMART TMB Peroxidase ELISA Substrate (Antibody Research Corporation) was added and incubated for 10 min at room temperature. Finally, reactions were terminated by addition of 100 μl 12.5% H_2_SO­_4_, and plates were read at 450 nm using a Synergy HTX Multi-Mode Reader (BioTek, Winooski, VT). Wells with no InvL added (PBS only) served as blanks, two technical and biological replicates were performed, and nonlinear regression analyses in GraphPad version 9 (GraphPad Software, San Diego, CA) was used to determine K_d_s.

**Digestion of secretome samples.** Precipitated secretomes were resuspended in 6 M urea and 2 M thiourea in 40 mM NH_4_HCO_3_ and then reduced for 1 h with 20 mM DTT. Reduced samples were then alkylated with 50 mM of iodoacetamide for 1 h in the dark. The alkylation reaction was then quenched by the addition of 50 mM DTT for 15 min and samples digested with Lys-C (1/200 w/w) for 3 h at room temperature. Samples were diluted with 100 mM NH_4_HCO_3_ four-fold to reduce the urea/thiourea concentration below 2 M then trypsin (1/50 w/w) added and allowed to digest overnight at room temperature. Digested samples were acidified to a final concentration of 0.5% formic acid and desalted with home-made high-capacity StageTips composed of 1 mg Empore™ C18 material (3M, Saint Paul, MN) and 5 mg of OLIGO R3 reverse phase resin (ThermoFisher Scientific) as described (10, 11). Columns were wet with Buffer B (0.1% formic acid, 80% acetonitrile) and conditioned with Buffer A* (0.1% TFA, 2% acetonitrile) prior to use. Acidified samples were loaded onto conditioned columns, washed with 10 bed volumes of Buffer A*, and bound peptides were eluted with Buffer B before being dried then stored at -20˚C.

**LC-MS analysis of Secretome samples.** Dried secretome digests were re-suspended in Buffer A* and separated using a two-column chromatography set up composed of a PepMap100 C18 20 mm x 75 μm trap and a PepMap C18 500 mm x 75 μm analytical column (ThermoFisher Scientific). Samples were concentrated onto the trap column at 5 μL/min for 5 min with Buffer A (0.1% formic acid, 2% DMSO) and then infused into an Orbitrap Q-Exactive plus Mass Spectrometer (ThermoFisher Scientific) at 300 nl/min via the analytical column using a Dionex Ultimate 3000 UPLC (ThermoFisher Scientific). 125-min analytical runs were undertaken by altering the buffer composition from 2% Buffer B (0.1% formic acid, 77.9% acetonitrile, 2% DMSO) to 22% B over 95 min, then from 22% B to 40% B over 10 min, then from 40% B to 80% B over 5 min. The composition was held at 80% B for 5 min, and then dropped to 2% B over 2 min before being held at 2% B for another 8 min. The Q-Exactive plus Mass Spectrometer was operated in a data-dependent mode automatically switching between the acquisition of a single Orbitrap MS scan [375-1400 m/z, maximal injection time of 50 ms, an Automated Gain Control (AGC) set to a maximum of 3 x 10^6^ ions and a resolution of 70k] and up to 15 Orbitrap MS/MS HCD scans of precursors (Stepped NCE of 28%, 30% and 35%, a maximal injection time of 100 ms, an AGC set to a maximum of 2*10^5^ ions, and a resolution of 17.5k).

**Proteomic analysis**. Secretome samples were processed using MaxQuant [v1.6.17.0. (12)] and searched against the NCBI annotated *A. baumannii* UPAB1 proteome (NCBI Accession: PRJNA487603, 3750 proteins, downloaded 2020-3-10). A six-frame translation of the UPAB1 genome generated using the six-frame translation generator within Maxquant and the ATCC17978 proteome (Uniprot: UP000319385, 3627 proteins, downloaded 2014-11-16) was used to allow the use of Uniprot annotation information associated with ATCC17978 proteins. Searches were undertaken using “Trypsin” enzyme specificity with carbamidomethylation of cysteine as a fixed modification. Oxidation of methionine and acetylation of protein N-termini were included as variable modifications and a maximum of two missed cleavages allowed. To enhance the identification of peptides between samples, the Match between Runs option was enabled with a precursor match window set to 2 min and an alignment window of 20 min with the label free quantitation (LFQ) option enabled (13). The resulting outputs were processed within the Perseus (v1.6.0.7) analysis environment to remove reverse matches and common protein contaminates prior to further analysis (14). For LFQ comparisons, biological replicates were grouped, and data was filtered to remove any protein which was not observed in at least one group three times. Missing values were then imputed based on the observed total peptide intensities with a range of 0.3σ and a downshift of 2.5σ using Perseus. Student *t*-tests were undertaken to compare the secretome between groups with the resulting data exported and visualized using ggplot2 within R (15). Shown in Table 1 are the average results from two biological replicates, and, specifically, candidate effector proteins with predicted signal peptides were included.

**Immunofluorescence assay.** 1.20 x 10^5^ 5637 cells were plated in one ml RPMI-1640 media in a 24-well plate on a glass cover-slip overnight. Cells were infected with stationary cultured UPAB1 at an MOI of one for one h and washed via the same techniques used for eukaryotic cell adhesion assays (See Materials and Methods). Following the final wash, cells were fixed with 4% paraformaldehyde in PBS for 15 min at 37°C and washed three times in PBS. Cells were then blocked in immunofluorescence assay (IFA) blocking buffer [IFA wash buffer (PBS w/ 0.5% BSA) supplemented with 10% goat serum] for one h at room temperature with rocking. Following blocking, cells were washed three times in IFA wash buffer for five min at room temperature with rocking. Primary antibody (1:500 rabbit α-UPAB1 in IFA wash buffer) was used to detect extracellular bacteria with a stationary incubation for one h at 37°C (16). Cells were then washed as above and incubated with 1:500 α-rabbit Alexa Fluor 647 (Invitrogen) stationary for one h at 37°C to stain extracellular bacteria far red. Cells were subsequently washed/permeabilized with IFA wash buffer supplemented with 0.05% saponin, and primary antibody (1:500 rabbit α-UPAB1) in IFA wash buffer supplemented with 0.05% saponin was added for one h stationary at 37°C. Cells were then washed in IFA wash buffer supplemented with 0.05% saponin, and 1:500 α-rabbit Alexa Fluor 488 (Invitrogen), 1X DAPI (Invitrogen), and 1X phalloidin Alexa Fluor 594 (Invitrogen) in IFA wash buffer with saponin was added stationary for one h at 37°C to stain total bacteria green, nuclei blue, and actin red. Therefore, extracellular bacteria were double stained green and far-red, whereas intracellular bacteria were only stained green. Cells were then washed as above in IFA wash buffer supplemented with 0.05% saponin, and cover slips were mounted on slides using ProLong Glass Antifade Mountant (ThermoFisher Scientific, Waltham, MA). Cells were imaged using the Zeiss LSM880 laser scanning confocal microscope (Carl Zeiss AG, Oberkochen, Germany), and a Plan-Apochromat 63X DIC objective and ZEN black 2.1 SP3 software were used for image acquisition. Images were processed using ImageJ software (National Institutes of Health, Bethesda, MD) (17).

**Separation of *A. baumannii* supernatant soluble and insoluble fractions.** WCL and supernatant fractions from early-stationary phase cultured WT UPAB1 harboring pBAV::*invL*-*his_6­_* were processed via instructions in the Materials and Methods. To isolate the insoluble fraction from early-stationary phase cultured WT UPAB1 harboring pBAV::*invL*-*his_6­_*, cells were centrifuged at 5432 x *g* for 8 min at 4°C. Supernatant was poured into a separate tube, centrifuged again, and twice-centrifuged supernatant was filtered using a Stericup Filter Unit (MilliporeSigma, Burlington, MA) with a 0.22 μm cutoff to remove any remaining bacteria. The filtered supernatant was then ultracentrifuged at 288244 x *g* overnight at 4°C using a Beckman Coulter Optima L-100 XP Ultracentrifuge (Beckman Coulter, Brea, CA), and the pellet was resuspended in PBS, Laemmli buffer was added to a concentration of 1X, and the sample was boiled at 99°C for 10 min. Supernatant from the ultracentrifugation was TCA precipitated via instructions for bacterial supernatant in the Materials and Methods. Volumes representing equivalent amounts of ultracentrifugation separated material and post-ultracentrifugation supernatant were separated by SDS-PAGE, and immunoblots were performed as described in the Materials and Methods.

**REFERENCES**

1. Tucker AT, Nowicki EM, Boll JM, Knauf GA, Burdis NC, Stephen Trent M, Davies BW. 2014. Defining gene-phenotype relationships in *Acinetobacter baumannii* through one-step chromosomal gene inactivation. mBio 5:1–9.

2. Datsenko KA, Wanner BL. 2000. One-step inactivation of chromosomal genes in *Escherichia coli* K-12 using PCR products. Proc Natl Acad Sci U S A 97:6640–6645.

3. Le NH, Peters K, Espaillat A, Sheldon JR, Gray J, Venanzio G di, Lopez J, Djahanschiri B, Mueller EA, Hennon SW, Levin PA, Ebersberger I, Skaar EP, Cava F, Vollmer W, Feldman MF. 2020. Peptidoglycan editing provides immunity to *Acinetobacter baumannii* during bacterial warfare. Sci Adv 6.

4. Harding CM, Tracy EN, Carruthers MD, Rather PN, Actis LA, Munson RS. 2013. *Acinetobacter baumannii* strain M2 produces type IV pili which play a role in natural transformation and twitching motility but not surface-associated motility. mBio 4.

5. Carruthers MD, Nicholson PA, Tracy EN, Munson RS. 2013. *Acinetobacter baumannii* utilizes a type VI secretion system for bacterial competition. PLoS One 8.

6. Ducas-Mowchun K, de Silva PM, Crisostomo L, Fernando DM, Chao TC, Pelka P, Schweizer HP, Kumar A. 2019. Next Generation of Tn 7-Based Single-Copy Insertion Elements for Use in Multi- and Pan-Drug-Resistant Strains of *Acinetobacter baumannii*. Appl Environ Microbiol 85.

7. Kumar A, Dalton C, Cortez-Cordova J, Schweizer HP. 2010. Mini-Tn7 vectors as genetic tools for single copy gene cloning in *Acinetobacter baumannii*. J Microbiol Methods 82:296–300.

8. Bryksin A v., Matsumura I. 2010. Rational design of a plasmid origin that replicates efficiently in both gram-positive and gram-negative bacteria. PLoS One 5.

9. Nesta B, Spraggon G, Alteri C, Moriel DG, Rosini R, Veggi D, Smith S, Bertoldi I, Pastorello I, Ferlenghi I, Fontana MR, Frankel G, Mobley HLT, Rappuoli R, Pizza M, Serino L, Soriani M. 2012. FdeC, a novel broadly conserved *Escherichia coli* adhesin eliciting protection against urinary tract infections. mBio 3.

10. Ishihama Y, Rappsilber J, Mann M. 2006. Modular stop and go extraction tips with stacked disks for parallel and multidimensional Peptide fractionation in proteomics. J Proteome Res 5:988–994.

11. Rappsilber J, Mann M, Ishihama Y. 2007. Protocol for micro-purification, enrichment, pre-fractionation and storage of peptides for proteomics using StageTips. Nat Protoc 2:1896–1906.

12. Cox J, Mann M. 2008. MaxQuant enables high peptide identification rates, individualized p.p.b.-range mass accuracies and proteome-wide protein quantification. Nat Biotechnol 26:1367–1372.

13. Cox J, Hein MY, Luber CA, Paron I, Nagaraj N, Mann M. 2014. Accurate proteome-wide label-free quantification by delayed normalization and maximal peptide ratio extraction, termed MaxLFQ. Mol Cell Proteomics 13:2513–2526.

14. Tyanova S, Temu T, Sinitcyn P, Carlson A, Hein MY, Geiger T, Mann M, Cox J. 2016. The Perseus computational platform for comprehensive analysis of (prote)omics data. Nat Methods 13:731–740.

15. Wickham H. Ggplot2 : elegant graphics for data analysis.

16. di Venanzio G, Flores-Mireles AL, Calix JJ, Haurat MF, Scott NE, Palmer LD, Potter RF, Hibbing ME, Friedman L, Wang B, Dantas G, Skaar EP, Hultgren SJ, Feldman MF. 2019. Urinary tract colonization is enhanced by a plasmid that regulates uropathogenic *Acinetobacter baumannii* chromosomal genes. Nat Commun 10.

17. Schneider CA, Rasband WS, Eliceiri KW. 2012. NIH Image to ImageJ: 25 years of image analysis. Nat Methods 9:671–675.
