# Supplementary material for: InvL, an Invasin-Like Adhesin, Is a Type II Secretion System Substrate Required for Acinetobacter baumannii Uropathogenesis
Source: mBio. 2022 May 31;13(3):e00258-22. doi: 10.1128/mbio.00258-22 (PMC9245377; doi:10.1128/mbio.00258-22)
Supplement: TABLE S3 [file mbio.00258-22-s0005.docx]

**Table S3. Primers used in this study.**

| **Primer** | **Sequence** |
| --- | --- |
| P1 | AGCGATTGTGTAGGCTGGAGCTG |
| P2 | CATATGAATATCCTCCTTAGTTCCTATTCCG |
| 5’ F1 gspDKO | AGTGGTAACTCAGGTGAAGG |
| 3’ F1 gspDKO | CGAAGCAGCTCCAGCCTACACAATCGCTCATAACTCGCGATATTTCCTAACTC |
| 5’ F2 gspDKO | GGAACTAAGGAGGATATTCATATGTAGTAGCATGTTATGATTAATAAAAAATGAATAGG |
| 3’ F2 gspDKO | TATCGTTTACAAACGTACCATTCG |
| AB FdeC Fwd | AATAAAATGAGCTTTACCCG |
| AB FdeC P1 Rev | TCCAGCCTACACAATCGCTTCCCCAATCTTTCCTAAAGG |
| CD FdeC P2 Fwd | TAAGGAGGATATTCATATGTATAAAAAGAAGCAGCCTTTGAGC |
| CD FdeC Rev | AGAACAACCAACCAGTGC |
| 5’ pUC for gspD | GGATCCACTAGTGAGCTCATGC |
| 3’ pUC for gspD | AAGCTTGGGCCCGGTACC |
| 5’ gspD Comp | AGCTCACTAGTGGATCCTGCCTCTTAAAAAGCCAAGCG |
| 3’ gspD Comp | TACCGGGCCCAAGCTTCTACGGCGCTGTGCTTGG |
| Tn7 linear Rev | GGATCCACTAGTGAGCTCATGC |
| His-pTn7 linear Fwd | CACCACCATCATCACCACTAAAAGCTTGGGCCCGGTACCTC |
| prom FdeC Fwd | ATGAGCTCACTAGTGGATCCTATGAATCCTAATAAC |
| His-FdeC- Rev | TTAGTGGTGATGATGGTGGTGATTACCATTTGAACAGTTTGG |
| 5’ pBAV linear marker swap | TTTTAAATATAGGATTTCATTTTCTCCCACC |
| 3’ pBAV linear marker swap | TACCGAGCTCGCTTGGACTCC |
| 5’ Apr for pBAV | GGAGTCCAAGCGAGCTCGGTATCAGCCAATCGACTGGCG |
| 3’ Apr for pBAV v2 | GAAAATGAAATCCTATATTTAAAATCGGTTCGTAAACTGTAATGC |
| N-term HisTag Fwd (for pBAV/fdeC) | CATCATCACCATCACCACTGATAATACTAGTAGCGGCCGCTGC |
| 3’ pBAV for UPAB1 fdeC natprom | AAGTCTTAATCTCTACTGCAATCATAATTGTGGTTTCAAAATCG |
| 5’ UPAB1 fdeC natprom for pBAV | TTTGAAACCACAATTATGATTGCAGTAGAGATTAAGACTTTACTTGG |
| fdeC REV (for pBAV) | TCAGTGGTGATGGTGATGATGATTACCATTTGAACAGTTTGGATC |
| fdeC NdeI Fwd | TAAGAAGGAGATATACATATGAAAAGTATGTATAAAAAACAAC |
| fdeC His noEND Rev | GTGGTGGTGGTGCTCGAGATTACCATTTGAACAGTTTGG |
| pET-22b linear Fwd | CATCATCATCATCACCACCACCACCACCACTGAGATCC |
| pET-22b linear Rev | GTTGTTTTTTATACATACTTTTCATATGTATATCTCCTTCTTAAAGTTAAAC |
| fdeC NdeI Fwd v2 | CTTTAAGAAGGAGATATACATATGAAAAGTATGTATAAAAAACAACTTACCTTG |
| FdeC 10His Rev | GGTGGTGGTGATGATGATGATGCTCGAGATTACCATTTGAACAGTTTGG |
| pET-22b linear Rev v2 | ATGTATATCTCCTTCTTAAAGTTAAAC |
| 5’ fdeC-SS for pET-22B | CTTTAAGAAGGAGATATACATATGATTATGTTAGCAAGTTGTGGCGGG |
